# Live Imaging of Xwnt5A-ROR2 Complexes

**DOI:** 10.1371/journal.pone.0109428

**Published:** 2014-10-14

**Authors:** Veronika Wallkamm, Rene Dörlich, Karolin Rahm, Tina Klessing, Gerd Ulrich Nienhaus, Doris Wedlich, Dietmar Gradl

**Affiliations:** 1 Zoological Institute, Department of Cell and Developmental Biology, Karlsruhe Institute of Technology, Karlsruhe, Germany; 2 Institute of Applied Physics and Insitute of Toxicology and Genetics, Karlsruhe Institute of Technology, Karlsruhe, Germany; 3 Department of Physics, University of Illinois at Urbana-Champaign, Urbana, Illinois, United States of America; University of Texas Medical Branch, United States of America

## Abstract

Secreted molecules of the Wnt family regulate key decisions in embryogenesis and adult tissue homeostasis by activating a complex network of Wnt signaling pathways. Although the different branches of Wnt signaling have been studied for more than 25 years, fluorophore tagged constructs for live cell imaging of Wnt molecules activating the Wnt/β-catenin pathway have become available only recently. We have generated a fluorophore tagged Wnt construct of the *Xenopus* Wnt5a protein (Xwnt5A) with the enhanced green fluorescent protein (EGFP), Xwnt5A-EGFP. This construct activates non-canonical Wnt pathways in an endocytosis dependent manner and is capable of compensating for the loss of endogenous Xwnt5A in *Xenopus* embryos. Strikingly, non-canonical Wnt pathway activation was restricted to short-range signaling while an inhibitory effect was observed in transwell cell cultures taken as long-range signaling model sytem. We used our Xwnt5A-EGFP construct to analyze *in vivo* binding of Wnt5A to its co-receptor ROR2 on the microscopic and on the molecular level. On the microscopic level, Xwnt5A-EGFP clusters in the membrane and recruits ROR2-mCherry to these clusters. Applying dual-colour dual-focus line-scanning fluorescence correlation spectroscopy on dorsal marginal zone explants, we identified membrane tethered Xwnt5A-EGFP molecules binding to ROR2-mCherry molecules. Our data favour a model, in which membrane-tethered Wnt-5A recruits ROR2 to form large ligand/receptor clusters and signals in an endocytosis-dependent manner.

## Introduction

Secreted cysteine-rich Wnt molecules constitute a highly conserved family of growth factors which consists of 21 genes in vertebrates (see wnt homepage at: http://www.stanford.edu/group/nusselab/cgi-bin/wnt/). Wnt proteins activate different signaling cascades, including the Wnt/β-catenin, Wnt-Calcium and Wnt planar cell polarity pathways. These Wnt triggered pathways interact on several levels of signal transduction to specify the cellular response to any given ligand and/or ligand combination. Thus, they should rather be considered as a Wnt-signaling network [1], [2]. Common to all Wnt pathways is the binding of a ligand to seven-pass transmembrane receptors of the frizzled (Fz) family and the regulation of the intracellular adapter protein dishevelled (dsh). The x-ray structure of the Xwnt8/Fz-CRD complex revealed that Wnts interact with the cysteine-rich extracellular domain (CRD) of Fz *via* two hydrophobic interaction sites [3]. Importantly, the interaction sites of the Wnt ligand, the fatty acid modification and the cysteine-rich C-terminus are highly conserved among all Wnt proteins, including those activating non-canonical pathways. The decision which of the Wnt pathways is activated depends not only on the Wnt/Fz interaction but also on the recruitment of co-receptors [4]. To activate the Wnt/β-catenin pathway, binding of a canonical Wnt (Wnt1, Wnt3A or Wnt8) results in phosphorylation of the low density lipoprotein receptor related protein (lrp5/6) co-receptor to form a signalosome [5]. The receptor complexes are internalized in a caveolin and RAB8B dependent manner [6], [7] and translocated together with dsh and glycogen synthase kinase 3β (GSK3β) in multivesicular bodies [8]. As a consequence, the phosphorylation of cytoplasmic GSK3β targets including β-catenin is reduced. Hypophosphorylated β-catenin escapes the proteasome degradation machinery, accumulates in the cytoplasm and the nucleus and interacts with Tcf/Lef transcription factors to regulate the expression of β-catenin dependent Wnt target genes.

Much less is known about the activation of non-canonical Wnt pathways that regulate planar cell polarity and convergent extension movements. Many of the coreceptors involved in non-canonical Wnt signal transduction are receptor kinases including PTK7 [9], Ryk [10] and ROR2 [11]. In either case, Wnt pathway activation should be considered as a highly dynamic process involving the clustering of signaling complexes and their internalization. For an in-depth analysis, fluorescence microscopy using fluorophore-tagged Wnt ligands is a powerful technique. However, the addition of tags, like myc-tags and EGFP-tags, often results in fusion proteins that have lost their biological function. Only recently, two biologically active fluorescently tagged Wnts have been reported, zWnt8-EGFP [12] and Xwnt2B-EGFP [13]. Both activate the canonical Wnt/β-catenin pathway. Due to their fatty acid modification, Wnt proteins are highly hydrophobic and difficult to purify. This also holds true for fusions of Wnts and fluorescent proteins. Indeed, live cell imaging of Xwnt2B-EGFP and zWnt8-EGFP revealed that these proteins are mainly found in the Wnt producing cells and in their direct neighbours. For non-canonical Wnts, an active fluorescently tagged construct able to compensate for the loss of the endogenous Wnt has not yet been reported.

Here we have generated an EGFP tagged version of Xwnt5A that is active in the non-canonical Wnt-responsive ATF2 reporter gene assay and in *Xenopus* embryos. Reconstitution experiments revealed that it can replace endogenous Xwnt5A in a specific non-canonical Wnt assay, the elongation assay of dorsal marginal zone explants. Reporter gene assays in cell culture revealed that cotransfected short-range Wnt5A-EGFP activates the non-canonical ATF2-Luc reporter in an endocytosis dependent manner. Dual-colour dual-focus line-scanning fluorescence correlation spectroscopy demonstrates membrane localization of Xwnt5A-EGFP and *in vivo* binding of Wnt5A and ROR2 in the membrane of the receptor expressing cell. ROR2-recruitment upon Wnt5A expression and ligand/receptor complex clustering in the plasma membrane further confirms the biological activity of both fusion proteins and thus their potential as tools to study Wnt5A signaling *in vivo*.

## Results

**Figure 1:**
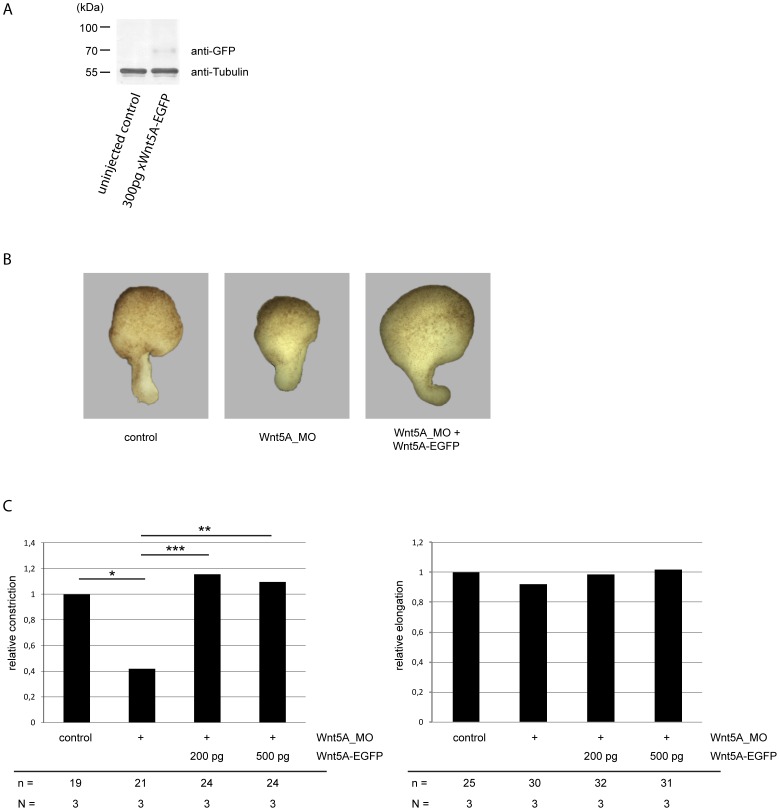
Xwnt5A-EGFP can compensate for the loss of endogenous Xwnt5A. A) An anti-EGFP reactive protein of the expected size is produced by embryos injected with Xwnt5A-EGFP. 300 pg Xwnt5A-EGFP mRNA were injected into each blastomere of two-cell stage embryos. NOP lysates corresponding to one half embryo were separated on an 8% SDS PAGE, transferred onto nitrocellulose and incubated with an anti-EGFP and anti-tubulin antibody. B) 1.6 pmol Xwnt5A morpholino (5AMo) were injected together with the indicated amounts of Xwnt5A-EGFP mRNA in the two dorsal blastomeres of eight-cell stage embryos. At stage 10.25, dorsal marginal zones were explanted and analyzed for convergence and extension. C) Quantification of elongation and constriction phenotypes. Given is the portion of embryos with impaired constriction (left image) and impaired elongation (right image) of Xwnt5A morphants (Wnt5A_Mo) and morphants co-injected with the indicated amount of Xwnt5A-EGFP mRNA. N: number of independent experiments, n: number of analyzed explants. *: p<0.05, **: p<0.01, ***: p<0.001 according to Fisher's exact test.

The addition of large tags to Wnt proteins may lead to misfolding or disturbance of their secretion resulting in biologically inactive Wnt proteins. To ensure that our fluorescently tagged Xwnt5A construct is biologically active, we injected mRNA of Xwnt5A-EGFP into *Xenopus* embryos. We subsequently observed production of a protein of the expected size (Fig. 1A). Degradation products were not visible, suggesting that the Xwnt5A-EGFP fusion protein is stable. To enquire if our fusion construct is also active *in vivo*, we tested whether it can compensate the loss of endogenous Xwnt5A. As a read-out system we used the elongation assay of *Xenopus* dorsal marginal zone (DMZ) explants [14]. As was shown previously [15], depletion of Xwnt5A by dorsal injection of antisense morpholino oligonucleotides results in a failure of convergent extension movements. Compared to the controls, the elongation of the explants was unaffected, whereas the explants of the morphants were much thicker (Fig. 1B). Due to Xwnt5A depletion the explants displayed a constriction phenotype. This effect was restored by simultanous injection of mRNA encoding for Xwnt5A-EGFP (Fig. 1C). Thus, Xwnt5A-EGFP can replace endogenous Xwnt5A and is, therefore, the first non-canonical fluorescent protein tagged Wnt construct proven to be active.

Next we asked, whether Wnt5A-induced signal transduction is due to autocrine and paracrine signaling, or whether also secreted Xwnt5A-EGFP might contribute to pathway activation in the form of long-range Wnt5A signaling.

**Figure 2:**
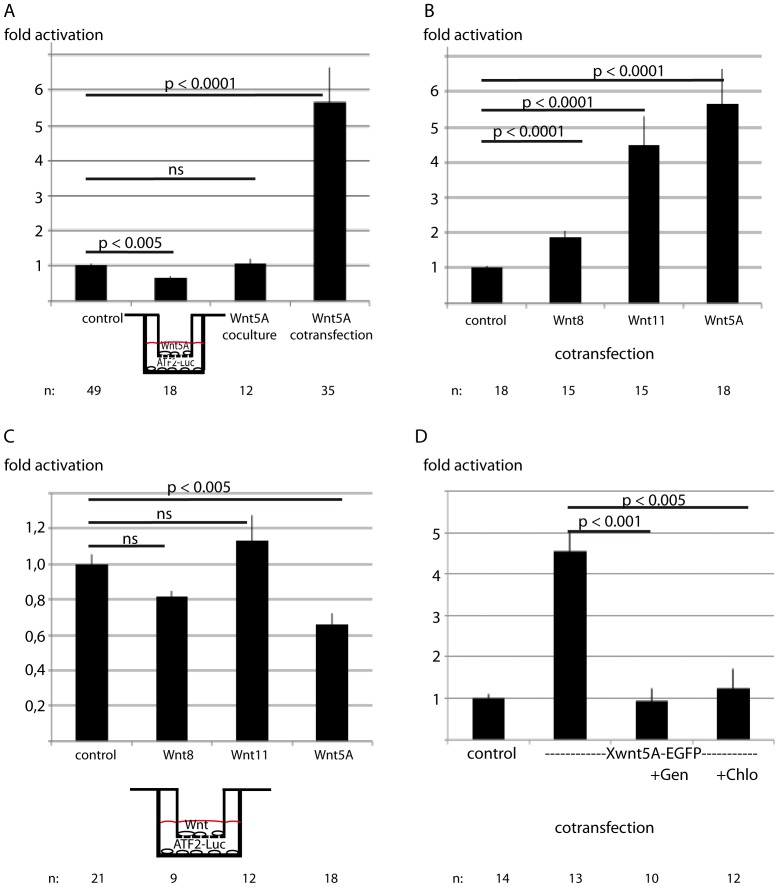
Long-range Wnt-5A inhibits the ATF2-Luc reporter. A) For studying long-range signaling, Xwnt5A-EGFP transfected HEK293 cells seeded on thinCerts TC chambers were transferred on cells transfected with the ATF2-Luc reporter and cultivated for additional 24 hours. To investigate paracrine signaling, cells transfected with Xwnt5A-EGFP were co-cultured with cells transfected with the ATF2-Luc reporter for 24 hours. To analyze both autocrine and paracrine signaling, the reporter and the Wnt ligand were cotransfected. B) Activation of the non-canonical Wnt reporter ATF2-Luc by co-transfected Wnt8, Wnt11 and Wnt5A. C) Activation of the non-canonical Wnt reporter ATF2-Luc by long-range Wnt8, Wnt11 and Wnt5A in a two-chamber assay. D) Xwnt5A-EGFP triggered ATF2-Luc activation depends on endocytosis. Wnt5A induced ATF2-Luc activation (cotransfection) was inhibited by addition of 5 µg/ml chlorpromazine (Chlo) and 150 µM genistein (Gen) 24 h before the measurement. Given are the mean values ± standard errors and the p-values according to Student's t-test, ns: not significant, n gives the number of transfections.

To monitor non-canonical Wnt signaling we used the ATF2-Luc construct as a read-out system for non-canonical Wnt driven transcription [16]. Co-transfected Xwnt5A-EGFP activated the ATF2-Luc reporter construct more than five fold. To explore if this robust activation was due to autocrine or paracrine Wnt5A signaling, we transfected Xwnt5A-EGFP and the reporter separately and co-cultured the transfectants for 24 hours. Under these conditions a physical contact of Wnt producing and receiving cells is given but autocrine signaling is excluded. Sensing paracrine and long-range signaling in this experiment we found no activiation of the reporter (Fig. 2A). Thus, most of the signaling activity derives from the autocrine Xwnt5A-EGFP. However, long-range Wnt5A slightly inhibits non-canonical Wnt signaling in the two chamber assay and, thus in a situation where we can clearly exclude a physical contact between the Wnt producing and the Wnt receiving cell. This inhibition was found only for Wnt5A. Wnt11 strongly activated the ATF2-Luc reporter in cotransfectants (Fig.2B) but did not regulate the promoter activity in the two-chamber assay (Fig.2C). Wnt8 activates the ATF2-Luc only less than two-fold in the cotransfectants and seems to decrease the promoter activity in the „long-range assay“, however this could not be confirmed by p-value calculation (Fig.2B, C).

To verify whether internalization is essential for Wnt5A induced pathway activation, we incubated the transfectants with the endocytosis inhibitors genistein and chlorpromazine and measured the activaty of the ATF2-Luc reporter. Indeed, both inhibitors significantly blocked Wnt5A-mediated reporter gene activation (Fig. 2D), indicating that internalization of Wnt5A is required for pathway activation. This points to a secretion of the ligand and extracellular binding to the receptor in autocrine signaling.

The low number of Wnt5A molecules responsible for the inhibitory long-range signaling are currently below the detection limit of our live-cell imaging set-up, so we focused on autocrine and paracrine signaling. Similar to Xwnt2B-EGFP [13], most of the Xwnt5A-EGFP remained in the endoplasmic reticulum (ER) of the producing cell and was concentrated in highly mobile vesicles and at the very end of long filopodia (Figure S1). These mobile fluorescent spots point to freshly produced Xwnt5A-EGFP molecules and/or molecules signaling back on the Wnt producing cell in an autocrine manner. Colocolization with early endosomal antigene (EEA) and caveolin1 (Figure S2) support the idea, that part of the intracellular Wnt was taken up from the outside.

To analyze ligand-receptor interaction, we chose DMZ explants of *Xenopus* embryos injected with our Wnt5A-EGFP construct because this tissue is known to require endogenous Wnt5A signaling (Fig. 1C, [15]). Thus, the subcellular localization of fluorophore tagged Wnt5A most likely reflects the localization of endogenous Wnt5A molecules. Furthermore, in these explants, the background from our construct in the cytosol and ER was much lower. We used these DMZ explants to analyze the mobility of individual ligand and receptor molecules at the membrane by dual-colour dual-focus line-scanning fluorescence correlation spectroscopy (2c2f lsFCS).

In a conventional FCS experiment, fluorescent molecules diffuse freely through an observation volume in a confocal microscope that is held at a fixed position. The fluctuations in fluorescence intensity are continously monitored, so that the intensity autocorrelation function, G(τ) can be computed as a function of the lag time τ. The fluorophore concentration and the diffusion coefficient can be determined from the amplitude and the characteristic decay time of G(τ), respectively. For this analysis however, the size of the observation volume has to be precisely determined by a reference measurement. In dual-focus FCS intensity fluctuations are recorded from two observation volumes that are spatially separated by a small, yet known distance [17], [18]. Here, the concentration and diffusion coefficient can be extracted directly from the fit of a model function without a calibration experiment. In bimolecular binding experiments, the interactions of two fluorescently labeled molecules can be studied by dual-color fluorescence cross-correlation spectroscopy (FCCS). Fluorescence fluctuations from two spectral channels are cross-correlated ([18], and the cross-correlation function has only an appreciable amplitude if two fluorescent particles diffuse as an entity, i.e., if they are bound to each other.

The static FCS approach is not easily applicable for measuring receptor-ligand interactions in cell membranes. Membrane movements also give rise to fluorescence fluctuations which can completely mask the fluctuations due to the diffusion of molecules within the lipid membrane. Line-scanning FCS was developed to overcome this problem [18], in which the observation volume is repeatedly scanned perpendicularly through a cell membrane. From the fluorescence intensity recorded while the observation volume intersects the membrane, the intensity time trace and intensity correlation functions can be calculated.

Here, we have used 2c2f lsFCS, a method that combines the two dual-focus line-scanning FCS calibration measurements for the red and green channel, respectively, and two dual-color line-scanning FCS measurements (red focus one with green focus one and red focus two with green focus two) to quantify bimolecular interactions in a single measurement. In addition, with the parallel measurement procedure we can also calculate dual-color dual-focus cross-correlations (red focus one with green focus two and red focus two with green focus one) to further enhance the statistics. By globally fitting all the data with a single set of parameters, this integrated approach combining two FCS modes yields very robust results.

**Figure 3:**
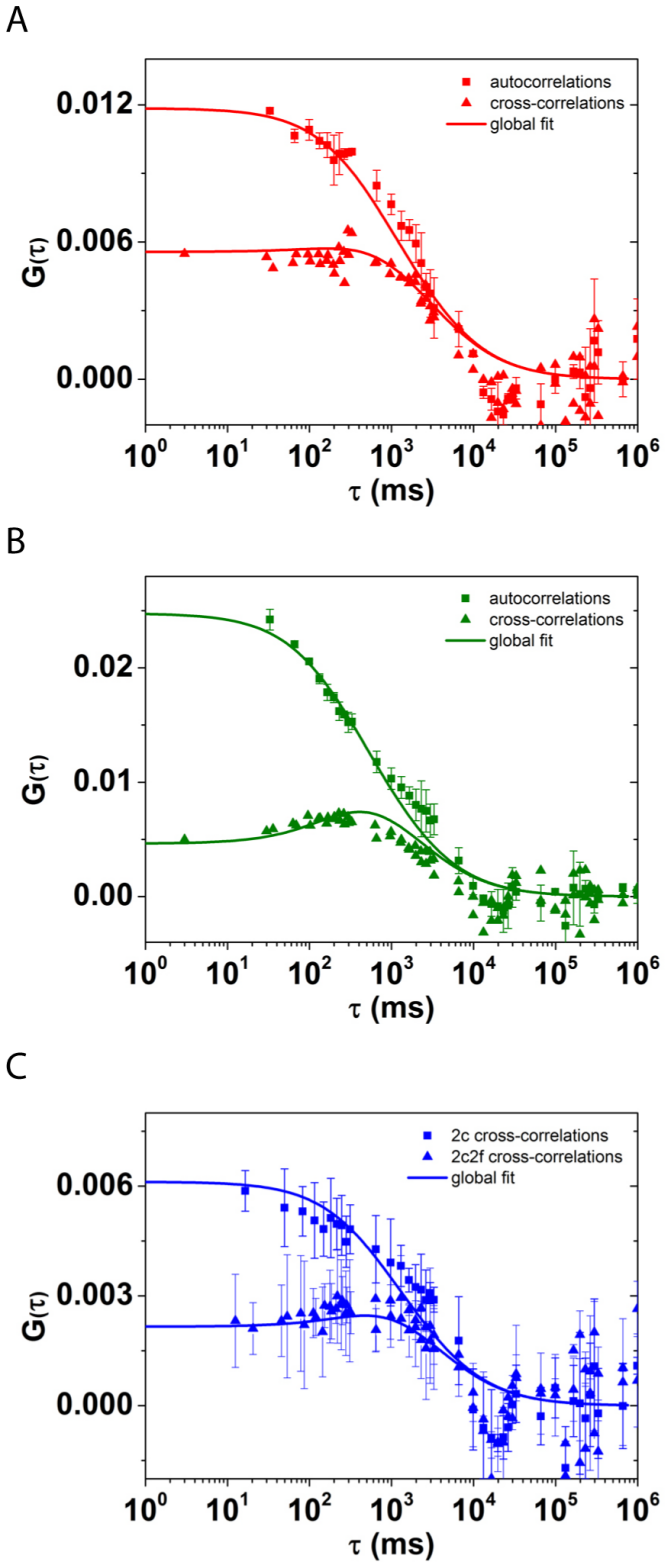
Fluorescence intensity correlation functions. A) Auto- and dual-focus cross-correlation functions in the red channel show ROR2-mCherry diffusion within the plasma membrane. B) Auto- and dual-focus cross-correlation functions in the green channel show the mobility of Xwnt5A-EGFP at the membrane. C) Cross-correlation functions of the green and red channels, reveal concerted diffusion and, therefore, binding of Xwnt5A-EGFP to ROR2-mCherry.

The auto- and dual-focus cross-correlation functions in the red channel (Fig. 3A) are determined by the concentration (i.e., area density in two dimensions), *C*_R_ = 23±12 µm^−2^, and diffusion coefficient, *D*_R_ = 0.14±0.06 µm^2^s^−1^, of the ROR2-mCherry receptor in the membrane. For Xwnt5A-EGFP, the auto- and cross-correlation curves (Fig. 3B) yielded a similar diffusion coefficient, *D*_L_ = 0.14±0.03 µm^2^s^−1^, and concentration, *C*_L_ = 23±5 µm^−2^. The cross-correlation function of the ROR2-mCherry and Xwnt5A-EGFP fluctuations (Fig. 3C) indicates significant binding between Xwnt5A-EGFP and ROR2-mCherry. Compared with the ligand and receptors alone, the diffusion coefficient of the ligand/receptor complex is smaller, *D*_RL_ = 0.07±0.02 µm^2^s^−1^, which is expected for the larger complex. The concentration of ligand/receptor complexes at the membrane, *C*_RL_ = 15±3 µm^−2^, suggests that approximately 40% of Xwnt5A-EGFP and ROR2-mCherry molecules are bound within a ligand/receptor complex.

**Figure 4:**
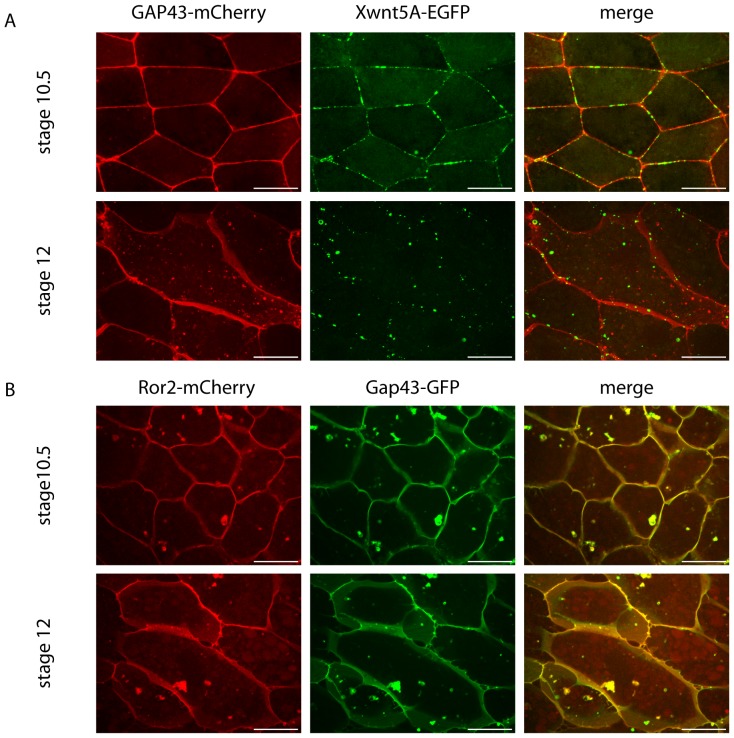
Xwnt5A-EGFP but not ROR2-mCherry clusters in membranes of DMZ cells. A) 150 pg of Xwnt5A-EGFP mRNA were co-injected with 125 pg membrane-anchored (growth-associated protein 43 (Gap43))-mCherry as a lineage tracer in the two dorsal blastomeres of eight-cell stage embryos. At stage 10.25, dorsal marginal zones were explanted and analyzed for subcellular localization of the Xwnt5A-EGFP protein. Prominent Xwnt5A-EGFP clusters localized at the membrane of DMZ cells at the onset (stage 10.5) and end (stage 12) of gastrulation. Shown are snapshots of movies S1 and S2. B) 40 pg ROR2-mCherry were injected into the two dorsal blastomeres of eight-cell stage embryos. ROR2-mCherry is homogenously distributed at the membrane of DMZ explants throughout gastrulation. Shown are snapshots of movies S3 and S4.

Consistent with the FCS data, we found Xwnt5A-EGFP located most prominently at the plasma membrane of DMZ explant cells (Fig. 4A). Interestingly, Xwnt5A-EGFP was not homogeneously distributed over the entire membrane, but formed bright mobile clusters on the cell surface (movies S1, S2, S8). Some intracellular Xwnt5A-EGFP particles appeared also in the cytosol. It remains elusive whether these particles consisted of internalized or freshly synthesized protein. Also, mCherry tagged ROR2 stained the membrane of DMZ explant cells (Fig. 4B). Again, this membrane localization was stable over time (movies S3, S4, S9). In contrast to the ligand, the ROR2 co-receptor was distributed homogeneously over the entire plasma membrane.

**Figure 5:**
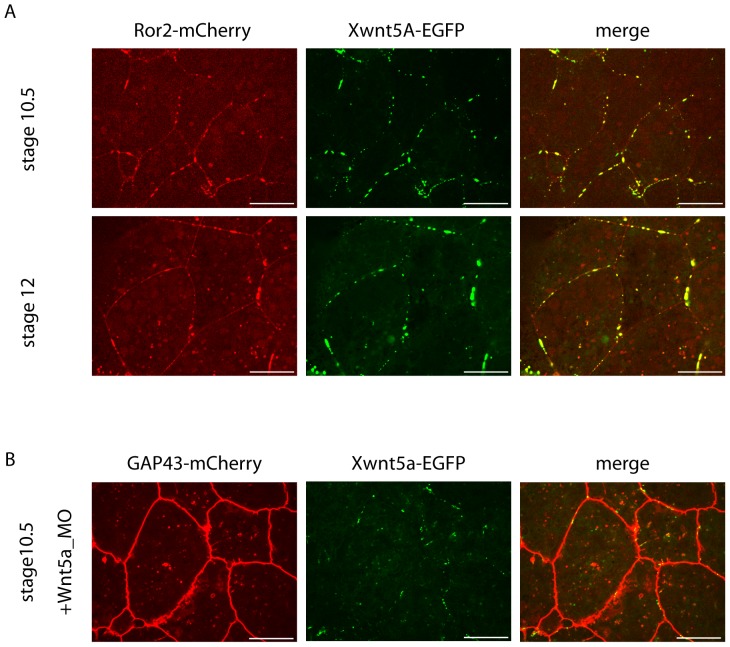
Xwnt5A induces clustering of ROR2. A) 150 pg of Xwnt5A-EGFP mRNA were co-injected with 40 pg of the ROR2-mCherry in the two dorsal blastomeres of eight-cell stage embryos. At stage 10.25 dorsal marginal zones were explanted and analyzed for subcellular localization of the Xwnt5A-EGFP and ROR2-mCherry. Prominent Xwnt5A-EGFP clusters co-localize with ROR2-mCherry clusters at the membrane of DMZ cells. These clusters are found at the onset (stage 10.5) and at the end (stage 12) of gastrulation. Shown are snapshots of movies S5 and S6. B) 150 pg of Xwnt5A-EGFP mRNA were co-injected with 1.6 pmol Xwnt-5A specific antisense morpholino and 125 pg Gap43-mCherry. Also in these Xwnt-5A-depleted explants Xwnt5A-EGFP clustered in the membrane.

However, when we co-injected Xwnt5A-EGFP together with ROR2-mCherry, the co-receptor co-clustered with the ligand at the membrane, indicating that Wnt5A recruits ROR2 to the membrane clusters (Fig. 5A). Again, these ligand/co-receptor clusters were stable over time, moved laterally in the membrane and occasionally appeared in the cytoplasm (movies S5, S6, S10).

To exclude that clustering in the membrane is simply an artifact of overexpressed Xwnt5A-EGFP, we repeated this experiment in Xwnt5A morphant embryos (Fig.5B and movie S7). In this experiment, the concentration of Xwnt5A-EGFP was high enough to compensate for the loss of endogenous Xwnt-5A, but not as high as to introduce effects on convergent extension movements (Fig. 1C). Similar to the overexpression experiment, Xwnt5A-EGFP was found in clusters at the cell membrane in this rescue experiment (Fig.5B and movie S7).

## Discussion

Here we report on a biofunctional Xwnt5A-EGFP construct that in HEK293 cells activates the ATF2-Luc reporter by short-range autocrine signaling while, long-range signaling studied by the transwell assay revealed an inhibitory effect of Xwnt5A-EGFP on the ATF2-Luc reporter. This inhibitory effect could also be a secondary effect of autocrine Wnt signaling, increasing the expression/secretion of Wnt inhibitors in the transfectants. To exclude that long range repression is restricted to overexpressed Wnt5A and the cell-culture system, an adequate *in vivo* assay has to be established.

Because the addition of endocytosis inhibitors efficiently blocked Wnt5A driven ATF2-Luc activation, we conclude that internalization of Wnt5A/ROR2 receptor complexes is essential for pathway activation. It has been speculated that similar to the Wnt3A induced caveolin-dependent internalization of lrp6 and Fz5 [19], a Wnt5A induced clathrin-dependent internalization of ROR might be essential for Wnt5A/ROR signaling [20]. In this scenario, the corecruitment of glypican4 to Wnt3A-induced caveolin-dependent complexes and to Wnt5A-induced clathrin-dependent complexes is involved in pathway regulation [21]. Similarily, O'Connell et al. [22] reported a clathrin-dependent internalization of ROR2 and showed immunohistochemically that both, ROR2 and Wnt5A localize in perinuclear spots. However, our own initial experiments indicated that inside a Wnt receiving cell, Xwnt5A-EGFP colocalizes with caveolin but not with clathrin (data not shown).

From our results, we can draw the important conclusion that the non-canonical Wnt reporter is activated mainly in an autocrine manner. Thus, in contrast to canonical Wnt signaling [23], long-range activation by non-canonical Wnts seems to be of minor relevance. Accordingly, we never observed Xwnt5A-EGFP more than one cell diameter apart from the producing cell (data not shown). This could simply result from lacking sensitivity, i.e., that we were unable to detect free Xwnt5A-EGFP on the single-molecule level outside the cells and at the membrane of non-Wnt producing cells. We encountered a similar problem with the canonical Xwnt2B-EGFP ligand earlier [13]. However, long-range repression of ATF2-Luc by Xwnt5A-EGFP indicates that at least this particular Wnt ligand is able to signal in a long-range manner.

Another important observation is that Wnt5A and Wnt5A/ROR2 complexes are not homogeneously distributed at the cell surface. Instead, Xwnt5A-EGFP clusters in the membrane of DMZ explants. A similar clustering of Wnt5A (but not of Wnt3A) was also observed in immunofluorescence studies in HeLa cells transfected with untagged Wnts [21]. Interestingly, the ligand Xwnt5A-EGFP forms clusters already in the absence of overexpressed ROR2. The overexpressed receptor, instead, is tethered into these clusters formed by Xwnt5A-EGFP. A Wnt5A-dependent clustering of ROR2 was recently demonstrated by Feike et al. [24], who incubated *Xenopus* animal cap tissue with Wnt5A-conditioned medium. Our results show that ROR2 and Wnt5A co-localize in these clusters. Together with the observation that Wnt5A activates the ATF2-Luc reporter only in a short-range manner, this might suggest a model in which Wnt5A sticks and clusters at the surface of the Wnt producing cell. These clusters recruit ROR2 and stay as ligand/receptor complexes at the membrane. To analyze the ROR2/Wnt5A complexes inside the clusters in more detail, the STED-RICS method [25], which has been applied recently to bright spots on plasma membranes of cultured cells, will be adapted to analyze *Xenopus* tissue in the future.

Distinct from these ligand/receptor clusters diffusing laterally in the membrane, we identified additional ligand/receptor complexes in the membrane between the large clusters by using dual-color dual-focus line-scanning fluorescence correlation spectroscopy (2c2f lsFCS) on DMZ explants of *Xenopus* embryos injected with ROR2-mCherry and Xwnt5A-EGFP. 2c2f lsFCS revealed that, although the concentration of Xwnt5A-EGFP and ROR2-mCherry in membrane areas between the clusters was very small (26 and 23 molecules per µm^2^, respectively), ligands and receptors efficiently formed complexes, as was clearly shown by the pronounced cross-correlation amplitude and the different diffusion coefficient of the ligand/receptor complex compared with the ligand and receptor alone. The concentration of ligand receptor complexes (15 molecules per µm^2^) indicate that, under these conditions, approximately 40% of ROR2 and Wnt5A molecules are engaged in ligand-receptor complexes. On the other hand, this means that about 60% of the membrane bound Wnt5A is not bound to ROR2. Whether these Wnt5A molecules are tethered to the membrane by their lipid modification or associated with other receptors including frizzled proteins remains presently elusive. However, whenever Wnt5A binds to ROR2, the size of the complex increases, as shown by a two-fold decrease of the diffusion coefficient.

Our data favour a scenario for Wnt5A induced non-canonical signaling where individual ROR2 molecules bind to individual membrane tethered Wnt5A molecules and contribute to the formation of large ligand/receptor complexes which diffuse laterally in the membrane and are internalized to activate the pathway.

## Methods

### Ethics

All animal studies were performed in strict accordance with German Animal Welfare legislation. All protocols and ethical evaluation were approved by the Institutional Animal Welfare Officer (Tierschutzbeauftragter) of the Karlsruhe Institute of Technology, and necessary licenses were obtained from the regional license granting body (Regierungspräsidium Karlsruhe, Germany; permit numbers: 35-9185.81/G-27/10). Necessary anesthesia was performed under MS-222 and all efforts were made to minimize suffering.

### Constructs and Inhibitors

ATF2-Luc [16], Xwnt11 [15], Xwnt8 [26], Wnt8-EGFP [27], ROR2-mCherry [24], Gap43-GFP [28] and Gap43-mCherry [28] were used as described earlier. To generate the Xwnt5A-EGFP construct, the open reading frame of Xwnt5A was combined by PCR with the open reading frame of EGFP and inserted in the XhoI site of pCS2. The Xwnt5A antisense Morpholino oligonucleotide was purchased from Gene Tools and used as described previously [11].

### Transfection and reporter assays

ATF2-Luc reporter gene assays were carried out as described [13]. To inhibit endocytosis, chlorpromazine and genistein were added to the samples 24 hours prior to the reporter measurements at a final concentration of 5 µg/ml and 150 µM, respectively. For co-culture experiments, cells were either transfected with reporter genes or Wnt constructs. 24 h after transfection, the transfectants were brought together and co-cultivated for another 24 hours. For “long-range assays”, 5×10^4^ HEK293 cells were seeded on thinCerts TC chambers with a pore size of 8.0 µm (Greiner Bio-One), transfected with 1 µg Xwnt5A-EGFP *via* Ca^++^-phosphate precipitation and cultivated in 1 ml DMEM medium supplemented with 10% FCS. 24 hours before analysis, the inlets with the transfected cells were transferred to a culture dish containing HEK293 cells transfected with ATF2-Luc and CMV-β-galactosidase.

### Injection and DMZ explants

Capped mRNAs were transcribed *in vitro* from linearized DNA templates using the mMESSAGE mMACHINE kit (Ambion). The embryos were staged according to Nieuwkoop and Faber, (1967). The injections for the DMZ explants and their explantation was described previously [28]. For microscopy, the explants are transferred into chamber slide coated with 1% BSA and fixed with a cover slip and analyzed with spinning disc microscopy (Observer Z1, Zeiss) and Axiovision 4.8.2 software (Zeiss) and ImageJ.

For the elongation assay, the explants were cultivated on a petri dish coated with 1% BSA until the sibling embryos reached stage 13. They were scored according to elongation. Only elongated explants were scored for constriction. For each explantation the explants were normalized to their uninjected sibling explants. Due to the binominial distribution and the total number of explants, significance was determined using Fisher's exact test.

### Western Blot

NOP lysates corresponding to one half embryo were separated on an 8% SDS PAGE, transferred to a nitrocellulose membrane, incubated with the primary antibodies anti-alpha tubulin (DM1A, Abcam) and anti-GFP (Abcam) and an alkaline phosphatase coupled secondary antibody [28].

### FCS

#### Microscopy Setup

FCS experiments were carried out on a home-built confocal microscope with fluorescence excitation at 488 and 561 nm by an Ar^+^ ion laser (Stabilite 2017, Spectra-Physics, Mountain View, CA) and a diode-pumped solid state laser (Jive, Cobolt AB, Sweden), respectively. The two laser beams were combined *via* a 540 nm long pass dichroic mirror (Q 540 LP, Chroma, Bellow Falls, VT). An acousto-optic tunable filter (AOTFnC-400.650, A-A Opto-Electronic, Orsay Cedex, France) was used for laser selection and intensity control. The excitation beam was circularly polarized by means of a quarter-wave plate (RAC 4.4.15, B-Halle, Berlin, Germany) so as to avoid photoselection of the fluorophores. After passing through a laser scanner (Yanus V, Till Photonics, Gräfelfing, Germany), the light was focused into the sample by using an oil immersion objective (HCX PL APO CS ×100/1.46, Leica, Wetzlar, Germany). The fluorescence emission was collected through the same objective, separated from the excitation light by a quad band dichroic mirror (zt405/488/561/640rpc, Chroma, Bellow Falls, VT, USA) and focused into a multimode fiber (M31L02; Thorlabs, Munich, Germany) serving as a confocal pinhole. Subsequently, the fluorescence light was separated by a 555 nm long pass dichroic mirror (Q 555 LP, Chroma) into two separate clour channels, which were spectrally filtered by a 525/50 (center/width) nm (Brightline HC 525/50, Semrock, Rochester, NY, USA) and a 600/37 nm bandpass filter (Brightline HC 600/37, Semrock). Photons were detected by avalanche photodiodes (tau-SPAD-50, PicoQuant, Berlin, Germany); their arrival times were registered by a data acquisition card (PCI-6259, National Instruments, Munich, Germany), which also provides the signals controlling the laser scanner and the acousto-optic tunable filter. Data acquisition parameters, including the number of pixels and lines, pixel sizes and dwell times are set *via* the software Imspector (Max-Planck-Innovation, Munich, Germany).

#### Data acquisition

For a single 2c2f lsFCS measurement, data were collected for 400 s. A single data acquisition cycle consisted of four line scans perpendicular to the membrane surface, i.e., scan with 488 nm in focus 1, scan with 488 nm in focus 2, scan with 561 nm in focus 1, scan with 561 nm in focus 2. Each scanned line consisted of 100 pixels with a width of 100 nm each, yielding a 10 µm scan range; the distance between the two scan lines was set to 400 nm.

#### Data Analysis

The analysis was performed with software written in Matlab (MathWorks, Natick, MA). The data of the four sequential scans were arranged in four two-dimensional arrays, with the pixels of each individual scan along the x-axis and the scanned lines sequentially along the y axis. To correct for membrane movements within the confocal volume, the membrane position within each line was determined by smoothing the data with a three-pixel averaging filter and identifying the maximum of the intensity. Subsequently, all peaks were shifted to the same column. The average over all scan lines was computed and fitted with a Gaussian function to determine the standard deviation (width parameter) σ. An intensity time trace was constructed by adding up, for each line, the pixel intensities within a range of ±2.5 σ from the center of the Gaussian. The auto- and cross-correlation curves of the four resulting intensity time traces were computed and globally fitted with model correlation functions by using a nonlinear least-squares fitting algorithm.

## Supporting Information

Figure S1 **Subcellular localization of Xwnt5A-EGFP.** XTC cells were transiently transfected with Xwnt5A-EGFP and Gap43-mCherry and analyzed as described [13]. Most of the overexpressed EGFP-tagged construct remains inside the wnt producing cell. The arrowheads point to a localization of Xwnt5A-EGFP at the tips of filopodia.
(TIF)

Figure S2 **Co-localization of Xwnt5A-EGFP with intracellular vesicles.** MDCK cells were transiently transfected with Xwnt5A-EGFP and analyzed by immunohistochemistry for colocalization with (A) caveolin, (B) clathrin 1 and (C) early endosomal antigene. A′, B′, and C′ are magnifications of A, B and C, respectively. Merge1 and merge2 show overlays with different contrast enhancements. Green arrows point to foci exclusively positive for Xwnt5A-EGFP, red arrows point to foci exclusively positive for the vesicle marker, yellow arrows indicate spots positive for both, Xwnt5A-EGFP and vesicle marker.
(TIF)

Movie S1 **Short time lapse sequence showing Xwnt5A-EGFP clusters moving in the membrane of DMZ explant cells (stage 10.5) and occasionally disappearing and appearing in the cytoplasm.** Optical sections (distance of 0.5 µm) were captured every 20 s for 2 min at room temperature.
(MOV)

Movie S2 **Xwnt5A-EGFP clusters move in the membrane of DMZ explant cells (stage 12) and occasionally disappear and appear in the cytoplasm.** Optical sections (distance of 0.5 µm) were captured every 20 s for 2 min at room temperature.
(MOV)

Movie S3 **Short time lapse sequence showing the localization of ROR2-mCherry in the membrane of DMZ explant cells stage 10.5.** Optical sections (distance of 0.5 µm) were captured every 20 s for 2 min at room temperature.
(MOV)

Movie S4 **Localization of ROR2-mCherry in the membrane of DMZ explant cells stage 12.** Optical sections (distance of 0.5 µm) were captured every 20 s for 2 min at room temperature.
(MOV)

Movie S5 **Short time lapse sequence showing that Xwnt5A-EGFP/ROR2-mCherry cluster move in the membrane of DMZ explant cells stage 10.5.** Optical sections (distance of 0.5 µm) were captured every 20 s for 2 min at room temperature.
(MOV)

Movie S6 **Xwnt5A-EGFP/ROR2-mCherry cluster move in the membrane of DMZ explant cells stage 12.** Optical sections (distance of 0.5 µm) were captured every 20 s for 2 min at room temperature.
(MOV)

Movie S7 **Tme lapse sequence showing Xwnt5A-EGFP clusters moving in the membrane of DMZ explant cells (stage 10.5) of Xwnt-5A morphant embryos.** Optical sections (distance of 0.5 µm) were captured every 20 s for 10 min at room temperature.
(MOV)

Movie S8 **Longer time lapse sequence showing Xwnt5A-EGFP clusters moving in the membrane of DMZ explant cells (stage 10.5).** Optical sections (distance of 0.5 µm) were captured every 20 s for 10 min at room temperature.
(MOV)

Movie S9 **Longer time lapse sequence showing ROR2-mCherry in the membrane of DMZ explant cells (stage 10.5).** Optical sections (distance of 0.5 µm) were captured every 20 s for 10 min at room temperature.
(MOV)

Movie S10 **Longer time lapse sequence showing Xwnt5A-EGFP/ROR2-mCherry clusters moving in the membrane of DMZ explant cells (stage 10.5).** Optical sections (distance of 0.5 µm) were captured every 20 s for 10 min at room temperature.
(MOV)
