# Supplementary figures and images for: Live Imaging of Xwnt5A-ROR2 Complexes
Source: PLoS One. 2014 Oct 14;9(10):e109428. doi: 10.1371/journal.pone.0109428 (PMC4196911; doi:10.1371/journal.pone.0109428)

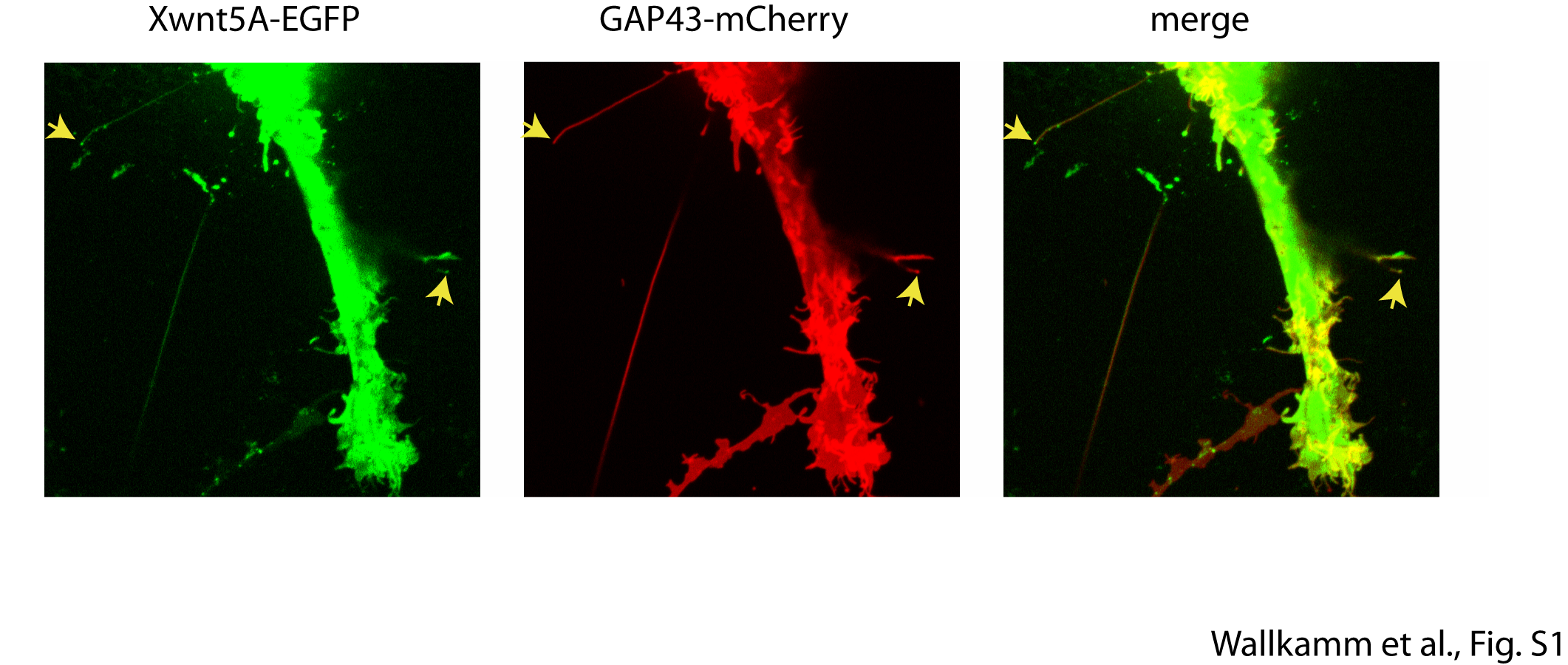

Supplement: Figure S1 — Subcellular localization of Xwnt5A-EGFP. XTC cells were transiently transfected with Xwnt5A-EGFP and Gap43-mCherry and analyzed as described [13]. Most of the overexpressed EGFP-tagged construct remains inside the wnt producing cell. The arrowheads point to a localization of Xwnt5A-EGFP at the tips of filopodia. (TIF) [file pone.0109428.s001.tif]

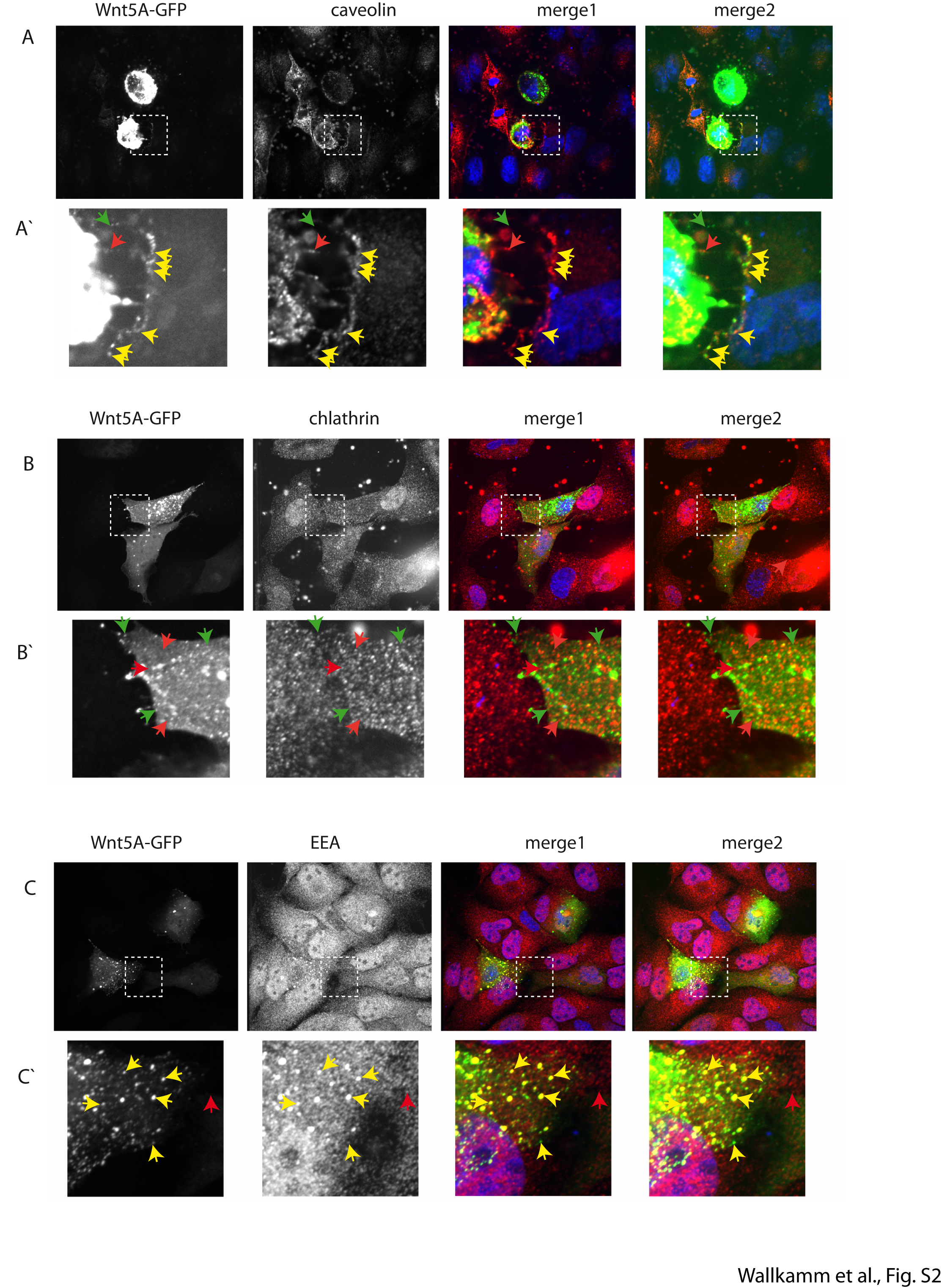

Supplement: Figure S2 — Co-localization of Xwnt5A-EGFP with intracellular vesicles. MDCK cells were transiently transfected with Xwnt5A-EGFP and analyzed by immunohistochemistry for colocalization with (A) caveolin, (B) clathrin 1 and (C) early endosomal antigene. A′, B′, and C′ are magnifications of A, B and C, respectively. Merge1 and merge2 show overlays with different contrast enhancements. Green arrows point to foci exclusively positive for Xwnt5A-EGFP, red arrows point to foci exclusively positive for the vesicle marker, yellow arrows indicate spots positive for both, Xwnt5A-EGFP and vesicle marker. (TIF) [file pone.0109428.s002.tif]
